# On the Value of Retaining the Roots of Teeth after the Loss of Their Crowns

**Published:** 1880-03

**Authors:** C. M. Wright

**Affiliations:** Basel, Switzerland


### ON THE VALUE OF RETAINING THE ROOTS OF TEETH AFTER THE LOSS OF THEIR CROWNS.

By C. M. Wright, Basel, Switzerland.

That there is an increased disposition on the part of dentists in the United States to make use of the roots of teeth, that have lost their crowns, may be noticed by the greater interest shown in methods of pivoting, methods of building out with bands and collars, around bicuspid and molar teeth, etc., as expressed in reports of the discussions of American Societies. Formerly our text books and instructors advised extraction where they would now advise us to retain and restore the crown. In many cases crowns have been almost entirely destroyed by neglect, by an acidity of the secretions of the mouth, by carelessness in the use of medicines and from other well known, or not well known causes, where the roots have remained firm and with a vigorous periodental vitality, in these cases extraction for the purpose of inserting artificial teeth upon the gums is not always indicated, the contour of the maxillary arch, the harmony of the features, the preservation of the agreeable expression of the face, and we are led to believe the health of the parts are better conserved by the retention of the roots, than by extraction and an after attempt at restoration by any artificial means; in the loss of a few of the crowns of incisors, cuspidati and bicuspid, the methods of pivoting artificial crowns upon the roots are generally successful, when carefully followed, and in cases of extensive destruction of the crowns, it is often well to prepare and fill the roots, dress them carefully with the corrundum wheels of the engine, and apply dental plates supporting artificial teeth over them.

I should like to describe a case that has been under my inspection for about seven years, constructed by an English dentist, the crowns of the superior wisdom teeth remain and are very solid and have never been attacked by caries or other disease, the cuspidati, (superior) and central incisors have been filled on their approximal surfaces with gold, the root and crown of one lateral incisor has been extracted, the roots of all the other nine teeth have been carefully dressed down with files, etc., and filled with amalgam. The arch is very large, broad and deep, and is covered by a stiff gold plate, nicely adapted over the roots and held in position by strong, narrow springy gold clasps adjusted to the wisdom teeth, single plain teeth, very thin, and presenting only the buccal surfaces, without having any grinding surfaces, have been arranged on the roots of the bicuspids and molars, and attached to the gold plate by strong, dark rubber, the rubber forming the grinding surface for the articulating with the lower teeth. The masticating ability offered by the hard rubber, being between the roots above and the teeth below is sufficient, the comfort is very great, the preservation of the contour quite perfect, the plate has been in hard use some ten or twelve years with an occasional renewal of the rubber.

The roots are all in a healthy condition, and the patient is satisfied that he can masticate better than if the case was constructed differently, on the grounds that in a grinding mill one of the stones is not so hard as the other.

Another case. Six years ago a lady called with the bicuspids and molars roots and crowns all missing, and the incisors and cupisdati in a wretched state of demoralization. Two or three had loose pivots held in the roots by cotton. The others were so broken that restoration seemed impracticable, especially as they had been pushed forward by mastication until they did not present a good appearance. The roots were long and firm. I cut off the remaining crowns, prepared and treated the roots, filled those that had large cavities caused by the loose pivots with amalgam, took impressions and set six pivot teeth. The pivots were of platinum wire soldered to small thin platinum plates that had been accurately adapted to the roots. The inclination of the pivots did not consequently prevent a proper inclination of the crowns that were of plate teeth, lined and soldered to the little plates that filled the roots. Holes of the proper size had been drilled through the hardened amalgam in the roots with the engine, and of the proper size to admit the platinum pivots which were finally cemented into the roots with shellac. An impression was then taken and the bicuspids and molars inserted on a rubber plate.

The lady is satisfied, for now as she expresses it, she can take her plate out and still have her front teeth left, avoiding the deformity that every one must deplore, of being toothless even when night draws her sable mantle round, &c.

In still other cases I have resorted to pivot teeth even where plates have been worn and the pleasure of the patients in being able to get rid of the nuisance of a dental plate has justified the operation, even if I had not felt inward satisfaction at the result, at having done something better than to cover up a palate with gold or rubber.

